# Transcription Factor EBF1 Over-Expression Suppresses Tumor Growth *in vivo* and *in vitro* via Modulation of the PNO1/p53 Pathway in Colorectal Cancer

**DOI:** 10.3389/fonc.2020.01035

**Published:** 2020-06-26

**Authors:** Zhiqing Shen, Youqin Chen, Li Li, Liya Liu, Meizhong Peng, Xiaoping Chen, Xiangyan Wu, Thomas J. Sferra, Meizhu Wu, Xiaoying Lin, Ying Cheng, Jianfeng Chu, Aling Shen, Jun Peng

**Affiliations:** ^1^Academy of Integrative Medicine, Fujian University of Traditional Chinese Medicine, Fuzhou, China; ^2^Fujian Key Laboratory of Integrative Medicine on Geriatrics, Fujian University of Traditional Chinese Medicine, Fuzhou, China; ^3^Department of Pediatrics, Case Western Reserve University School of Medicine, Rainbow Babies and Children's Hospital, Cleveland, OH, United States; ^4^Department of Health Management, Fujian Provincial Hospital, Fuzhou, China

**Keywords:** EBF1, colorectal cancer, survival, tumor growth, PNO1, p53 pathway

## Abstract

Early B cell factor 1 (EBF1) has been identified as an upstream transcription factor of the potential oncogene PNO1 and is involved in the growth of colorectal cancer (CRC) cells. However, its expression, biological function, and underlying mechanism of action in most solid tumors remain largely unknown. We postulated that EBF1 has a role in the pathophysiology of CRC. Analysis of EBF1 mRNA expression in CRC tumor samples from several public databases and directly from banked tissues revealed that EBF1 mRNA expression is lower in CRC tissue compared to non-cancerous colorectal tissue. Survival analysis of multiple datasets revealed that low EBF1 expression was correlated with shorter overall survival, relapse-free survival, and event-free survival in CRC patients. Transduction of lentivirus encoding full length EBF1 followed by *in vitro* and *in vivo* assays demonstrated that EBF1 over-expression in CRC cell lines suppresses cell growth by inhibiting cell viability, cell survival, and induces cell cycle arrest and apoptosis. Mechanistic investigation indicated that EBF1 over-expression down-regulates PNO1 mRNA and protein expression, as well as transcriptional activity while up-regulating the expression of p53 and p21 proteins. These findings suggest that EBF1 is a novel potential tumor suppressor in CRC with prognostic value for the identification of patients at high-risk of relapse.

## Introduction

Colorectal cancer (CRC) is one of the most common cancers with high morbidity and mortality worldwide (1–4). Despite the combination of current treatments for CRC including resection, radiotherapy, and chemotherapy, patient mortality remains high (5, 6). Therefore, it is urgently required to explore the underlying biology of CRC in order to inform the development of diagnostic, therapeutic, and prognostic targets and biomarkers.

The transcription factor Early B cell factor 1 (EBF1) is located on human chromosome 5q34 and expressed primarily in early B cells, adipocytes, and olfactory neurons (7, 8). EBF1 is involved in B cell (9, 10), bone (11), and kidney (12) development; B cell (13) and retinal cell (14) differentiation; and adipogenesis (15). Due to the essential role of EBF1 during B cell development, loss of EBF1 (16, 17) or an increase of one of its inhibitors [ZNF521 (18) or ZNF423 (19)] supports the development of B-cell acute lymphoblastic leukemia (B-ALL). Moreover, EBF1 regulates DNA repair in a dose-dependent manner by direct effects on RAD51, and combined loss of EBF1 and Pax5 predisposes patients to leukemia development (20, 21). These findings suggest a potential tumor suppressor role for EBF1 in leukemia.

Recent studies suggest EBF1 has a role in the pathogenesis of solid tumors. Decreased EBF1 expression resulting from genomic loss or somatic missense mutations were found in breast cancer (BC) (22, 23), pancreatic ductal adenocarcinoma (PDAC) (24), and cholangiocarcinoma (CCA) (25). Moreover, low levels of EBF1 correlated with shorter survival in patients with CCA (25). Pathophysiologic investigations demonstrated that EBF1 knockdown increases cell viability, wound healing, and cell migration, as well as the expression of CD133, Oct3/4 and TFF1 in CCA (25). In CRC, we identified EBF1 as an upstream transcription factor of PNO1 and found it suppressed CRC cell growth *in vitro* by negatively regulating PNO1 expression (26). However, the role of EBF1 in CRC has not been fully evaluated. Therefore, in the present study, the expression and prognostic significance of EBF1 in CRC patients was explored. Moreover, a lentivirus vector encoding full-length human EBF1 was used to investigate the role of EBF1 over-expression in the growth of CRC cells.

## Materials and Methods

### Bioinformatics Analyses

The following databases and analyses were used:

GEPIA: EBF1 mRNA expression in both colon and rectal cancers were analyzed using the GEPIA website (27–29) containing RNA sequencing and expression data from the TCGA and GTEx projects (http://gepia.cancer-pku.cn/detail.php). The median expression levels of EBF1 in both tumor tissues and non-cancerous tissues are presented in bar plots.

Oncomine: EBF1 expression in CRC and non-cancerous colorectal tissues was compared using the Oncomine database (www.oncomine.org) as described previously (26, 30–32). Data analysis was performed according to standardized normalization techniques and statistical calculations provided by the Oncomine website. The search parameters and filters were set as follows: *P*-value, 0.05; fold change, 1.5; gene rank, top 10%; analysis type, cancer vs. normal analysis; data type, mRNA.

R2 application: We explored the correlation between EBF1 expression and survival of CRC patients through the R2 application (http://r2.amc.nl) using the log-rank method in multiple datasets, including Mixed Colon Adenocarcinoma-TCGA-174-custom-agg4502a073, Tumor Colon MVRM-SieberSmith-345-Frma (b c)-u133p2, Tumor Colon MSI-status (Core-Transcript)-Sveen-95ma_sketch-huex10t, Tumor Colon (Core-Exon)-Sveen-333rma_sketch-huex10p and Tumor Colon MSI-status (Core-Exon)-Sveen-95rma_ sketch-huex10p). The cut-off was identified by methods described in the R2 web-based application (http://r2.amc.nl).

### Microarray Analysis

Microarray analysis was performed as previous described (26). Briefly, analysis the EBF1 expression was compared between colorectal cancer tissues (*n* = 14) and matched adjacent normal tissues (*n* = 14).

### Real-Time PCR Analysis and Tissue cDNA Array Analyses

Total RNA was isolated from cultured cell lines using RNAiso Plus reagent (Takara; Dalian, Liaoning, China) according to the manufacturer's instructions. cDNA was synthesized by RNA reverse transcription using PrimeScript RT kit (Takara). cDNA from 11 paired CRC samples was obtained from the cDNA-HColA095Su01 tissue cDNA array (Shanghai Outdo Biotech, Shanghai, China) and used to detect the mRNA levels of EBF1, PNO1, or GAPDH using an ABI 7500 Fast Real-Time PCR System (Applied Biosystems, Carlsbad, CA, USA) and the SYBR Premix Ex Tag (Takara). The relative mRNA expression was determined using the cycle threshold (CT) formula 2^−−ΔΔCT^, where ΔCT = [CT (target gene) – CT (GAPDH)]. The expression level was normalized against endogenous GAPDH. The specific primers were as follows: human EBF1, forward 5′-AGC TTC TCT ACA GCA ATG GGA T-3′ and reverse 5′-TGA GCA AGA CTC GGC ACA TT-3′; human PNO1, forward 5′-TGT TAA ACC CCT AAA GGG AGA CC-3′ and reverse 5′-CCT TGT CCG TGT CAC ATT CTC T-3′; human GAPDH, forward 5′-TGC ACC ACC AAC TGC TTA GC-3′ and reverse 5′-AGC TCA GGG ATG ACC TTG CC-3′.

### Cell Lines and Cell Culture

Human CRC cell lines HCT-116 and HT-29 were purchased from the Cell Bank of the Chinese Academy of Sciences (Shanghai, China) and were cultured in RPMI-1640 medium (Thermo Fisher Scientific, Waltham, MA, USA) supplemented with 10% fetal bovine serum (Thermo Fisher Scientific) and 1% penicillin-streptomycin (Hyclone, Logan, UT, USA). Cells were maintained in a humidified atmosphere at 37°C containing 5% CO_2_. Cells were verified using short tandem repeat genotyping and examined for mycoplasma contamination using RT-PCR.

### Generation of Stable Transduction Cell Lines

To generate a cell line stably over-expressing EBF1, HCT-116, or HT-29 cells were seeded in six-well plates (5 × 10^4^ cells/well) and transduced with a lentiviral vector encoding full-length human EBF1 (coding region of 756 bp; Shanghai, GeneChem) or empty vector for 72 h. Transduced cells were further selected for 2 weeks using puromycin (Thermo Fisher Scientific) at 1 μg/ml.

### Cell Confluence Observation and Cell Number Counting

Cells were seeded in 6-well plates (1 × 10^5^ cells/well) for 72 h and cell growth was assessed by observation of cell confluence using a phase contrast fluorescence microscope (Leica Microsystems; Wetzlar, Germany). Images were captured at a magnification of 200×. Cells were digested with trypsin and cell number was calculated by trypan blue exclusion using a Countstar Automated Cell Counter (Inno-Alliance Biotech, Inc.; Wilmington, DE, USA). Three independent experiments were performed, and data were normalized to results for control cells.

### Cell Viability Analysis

Cell viability was determined using the Cell Counting Kit-8 (CCK-8; Abbkine, Wuhan, Hubei, China). Briefly, cells were seeded into 96-well plates (1 × 10^3^/well) in complete medium and incubated at 37°C, 5% CO_2_. At specific time points (24–120 h), CCK-8 reagent (10 μl/well) was added into each well and incubated for an additional 2 h at 37°C in the dark. The absorbance was measured at 450 nm using a microplate reader (Bio-Tek, Winooski, VT, USA). The experiments were performed in three independent experiments in triplicate.

### Colony Formation Assay

Transduced cells were seeded in 12-well plates at a density of 500 cells per well and cultured with RPMI-1640 containing 10% FBS. After 8–10 days of culture, supernatants were discarded, and cells were washed with PBS, fixed with 4% paraformaldehyde for 20 min, and stained with 0.01% crystal violet (Solarbio, Beijing, China) for 20 min. Photographs were taken and colonies counted. Cell survival was calculated relative to that of control cells (set as 100% survival). The experiments were performed in triplicate.

### Cell Cycle Analysis

The stably transduced cells were harvested and washed with ice-cold PBS after the cells were re-seeded in 6-well plates and cultured for additional 72 h. Afterwards, cells were collected and fixed with 70% ice-cold ethanol overnight at 4°C. After fixation, the cells were washed with PBS twice and incubated with 400-μl of FxCycle PI/RNase Staining Solution (Thermo Fisher Scientific) at room temperature in the dark for 15 min according to the manufacturer's instructions. Cell cycle status was analyzed using by flow cytometry (Becton Dickinson, CA, USA). The experiments were performed three times.

### Cell Apoptosis Analysis

The cells stably transduced with the lentivirus vector were seeded in 6-well plates. After culture for 72 h, cells were incubated with Annexin V-APC solution (KeyGEN, Jiangsu, China) for 20 min according to the manufacturer's instructions. The percentage of apoptotic cells were analyzed by flow cytometry (Becton Dickinson). The experiments were performed in triplicate.

### Animal Experiment

All animal experiments were approved by the Institutional Animal Care and Use Committee at the Fujian University of Traditional Chinese Medicine and performed in strict accordance with the “Guide for the Care and Use of Laboratory Animals” and “Principles for the Utilization and Care of Vertebrate Animals.” Male BABL/c nude mice (6 weeks of age; 20–22 g) were purchased from Shanghai SLAC Laboratory Animal Co. (Shanghai, China). Mice were maintained in a specific pathogen-free facility. To assess the effect of EBF1 over-expression on tumor growth *in vivo*, the lentivirus vector stably transduced cells (EBF1 over-expressing and control) were injected subcutaneously into the opposite flanks of mice (*n* = 4). The injectate contained 1 × 10^6^ cells in RPMI1640 medium containing 50% Matrigel (Corning Life Science). Tumor volumes were measured with a micrometer caliper and calculated as 1/2 (length × width^2^) every 2 days from the fifth day after injection. At the end of the experiment, mice were anesthetized with isoflurane and euthanized, and tumors were collected, photographed, and weighed.

### Dual Luciferase Reporter Assay

To confirm the regulatory effect of EBF1 on PNO1 transcription, the stably transduced cells were transfected with a plasmid encoding firefly luciferase cDNA reporter downstream of a 2.0-kb fragment of the PNO1 promoter (500 ng) together with Renilla luciferase (20 ng) internal control plasmid using Lipofectamine 3000 Transfection reagent (Thermo Fisher Scientific). After 48 h in culture, the relative luciferase activity was determined using a dual-luciferase reporter assay kit (Promega, USA). Briefly, cells were washed with PBS twice and lysed with 1× Passive Lysis Buffer. The lysates were centrifuged and supernatants collected. Equal volumes of supernatant (20 μl) were added to 96-well plates, follow by 100 μl of luciferase assay reagent (Promega), The firefly luciferase activity was normalized to the Renilla luciferase activity and calculated relative to that of control cells set as 100%.

### Western Blotting

Cells were lysed with lysis buffer (Beyotime, Shanghai, China) containing PMSF (Amresco, Solon, Ohio, USA) for 30 min on ice, centrifuged at 12,000 g for 15 min at 4°C, supernatants collected, and total proteins measured using the BCA Protein Assay Kit (Thermo Fisher Scientific). Equal amounts of protein were separated on 10% sodium dodecyl sulfate polyacrylamide gel and transferred onto a 0.45 μm PVDF membrane (Millipore, Billerica, MA, USA). Membranes were then blocked with TBST containing 0.5% bovine serum albumin (BSA; Amresco, Solon, Ohio, USA), followed by incubation with the primary rabbit anti-EBF1 (1:1,000 dilution; Abcam, USA), rabbit anti-PNO1 (1:1,000 dilution; LSBio, USA), rabbit anti-p53 (1:1,000 dilution, Proteintech, USA), rabbit anti-p21 (1:1,000 dilution, CST, USA), or rabbit anti-GAPDH (1:1,000 dilution; CST, USA) antibodies overnight at 4°C. After washing with TBST, membranes were incubated with goat anti-rabbit secondary antibody at room temperature for 1 h and washed with TBST. Target proteins were detected using ECL kits (Thermo Fisher Scientific). The protein levels were analyzed with Image J. GAPDH protein expression was used as an internal control.

### Statistical Analysis

Data are expressed as the means ± standard deviations of at least three independent experiments. Differences between two groups were analyzed using unpaired or paired Student *t*-tests. All statistical analyses were performed using SPSS20.0 software and differences were considered significant when *P* < 0.05.

## Results

### EBF1 Is Down-Regulated in Human Colorectal Cancer

**Figure 1:**
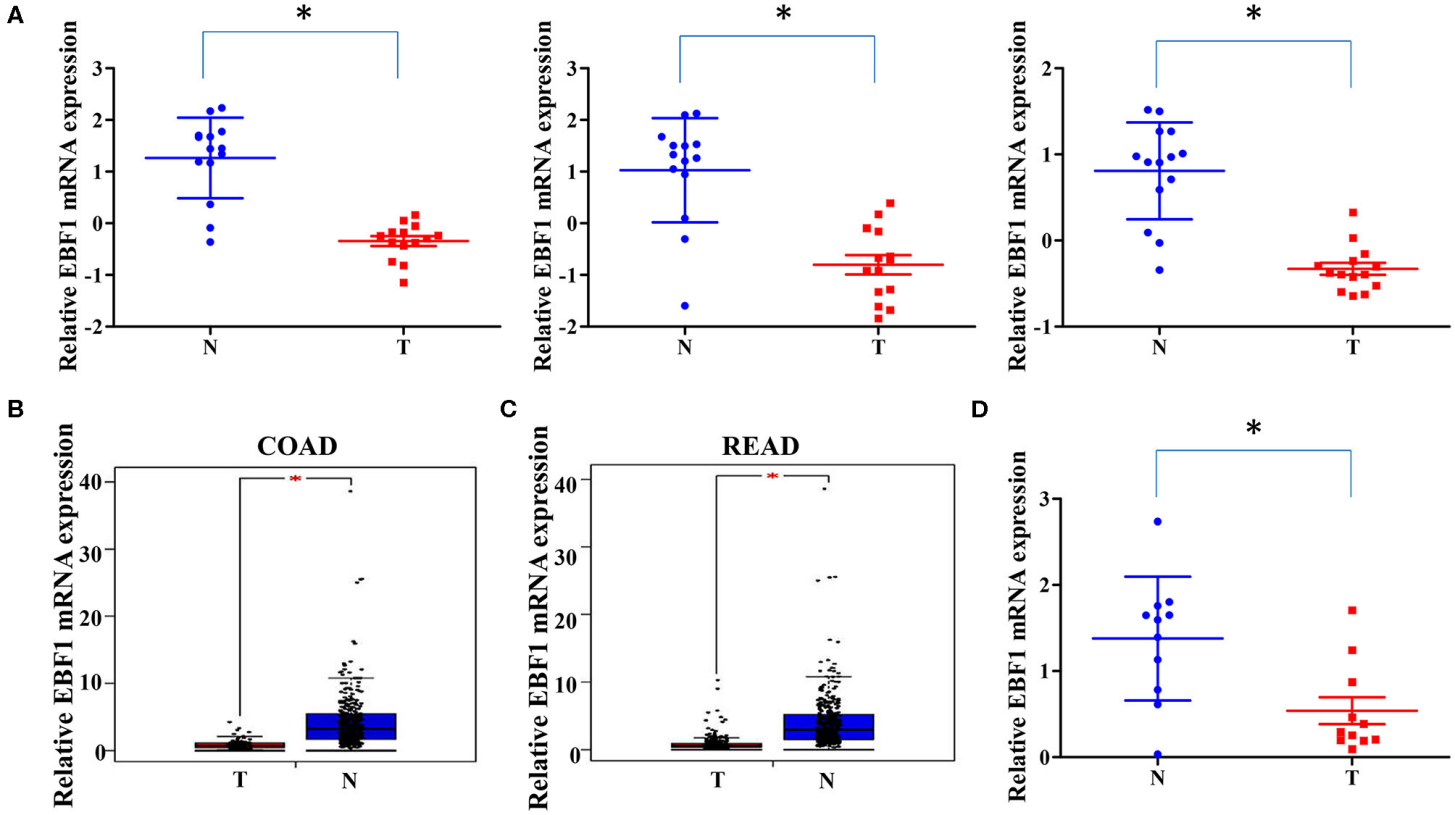
EBF1 is down-regulated in human colorectal cancer. **(A)** EBF1 mRNA expression (probe1: 11726800_at; probe2: 11726801_at; probe3: 11726802_a_at) in 14 matched CRC and non-cancerous colorectal tissues from our previously described gene expression profile microarrays (GEO Submission: GSE113513). Each dot represents 1 tissue. **(B,C)** EBF1 mRNA expression in **(B)** COAD tissues (*n* = 279) and normal colon tissues (*n* = 345), **(C)** READ tissues (*n* = 92) and normal rectum colon tissues (*n* = 318) were analyzed based on that in the TCGA datasets through GEPIA website. **(D)** EBF1 mRNA expression was determined in 11 pairs of CRC samples using Q-PCR and was normalized to GAPDH. T, colorectal cancer tissues; N, non-cancerous colorectal tissues. *P < 0.05 vs. N.

To investigate the expression of EBF1 in CRC, we first compared EBF1 expression in 14 matched pairs of primary CRC tumors and non-cancerous tissues from a cDNA array (GEO Submission: GSE113513). Consistent with our previous results (26), we found that EBF1 mRNA levels were lower in CRC compared to non-cancerous tissues (Figure 1A). Analysis of datasets within the GEPIA (Figures 1B,C) and Oncomine (Table 1) websites indicated that the mRNA levels of EBF1 were significantly down-regulated in CRC tissues as compared to non-cancerous colorectal tissues. Using cDNA from 11 paired CRC and normal tissue samples and Q-PCR analysis, we further confirmed that mRNA expression of EBF1 was down-regulated in CRC (Figure 1D). These results suggest that EBF1 may act as a tumor suppressor in CRC.

**Table 1:** Oncomine analysis of EBF1 expression in colorectal cancer (total 4 colorectal cancer cohorts).

**Cohort no**.	**Cohort**	**Sample (*n*)**	**Fold-change**	***P*-value**
1	TCGA	Rectal mucinous adenocarcinoma (6) vs. normal (22)	1.944	3.51E-10
		Cecum adenocarcinoma (22) vs. normal (22)	−4.229	2.27E-13
		Colon mucinous adenocarcinoma (22) vs. normal (22)	−3.602	4.58E-10
		Rectosigmoid adenocarcinoma (3) vs. normal (22)	−3.399	1.30E-04
		Rectal adenocarcinoma (60) vs. normal (22)	−4.331	4.43E-14
		Colon adenocarcinoma (101) vs. normal (22)	−4.226	1.20E-12
7	Sabates-Bellver Colon	Colon adenoma (25) vs. normal (32)	−3.125	6.15E-07
		Rectal adenoma (7) vs. normal (32)	−4.309	2.00E-03
9	Kaiser Colon	Rectal mucinous adenocarcinoma (4) vs. normal (5)	−2.182	7.93E-04
10	Skrzypczak Colorectal	Colorectal adenocarcinoma (45) vs. normal (24)	−2.546	1.56E-06

### Low EBF1 Expression Is Associated With Shorter Survival in CRC Patients

**Figure 2:**
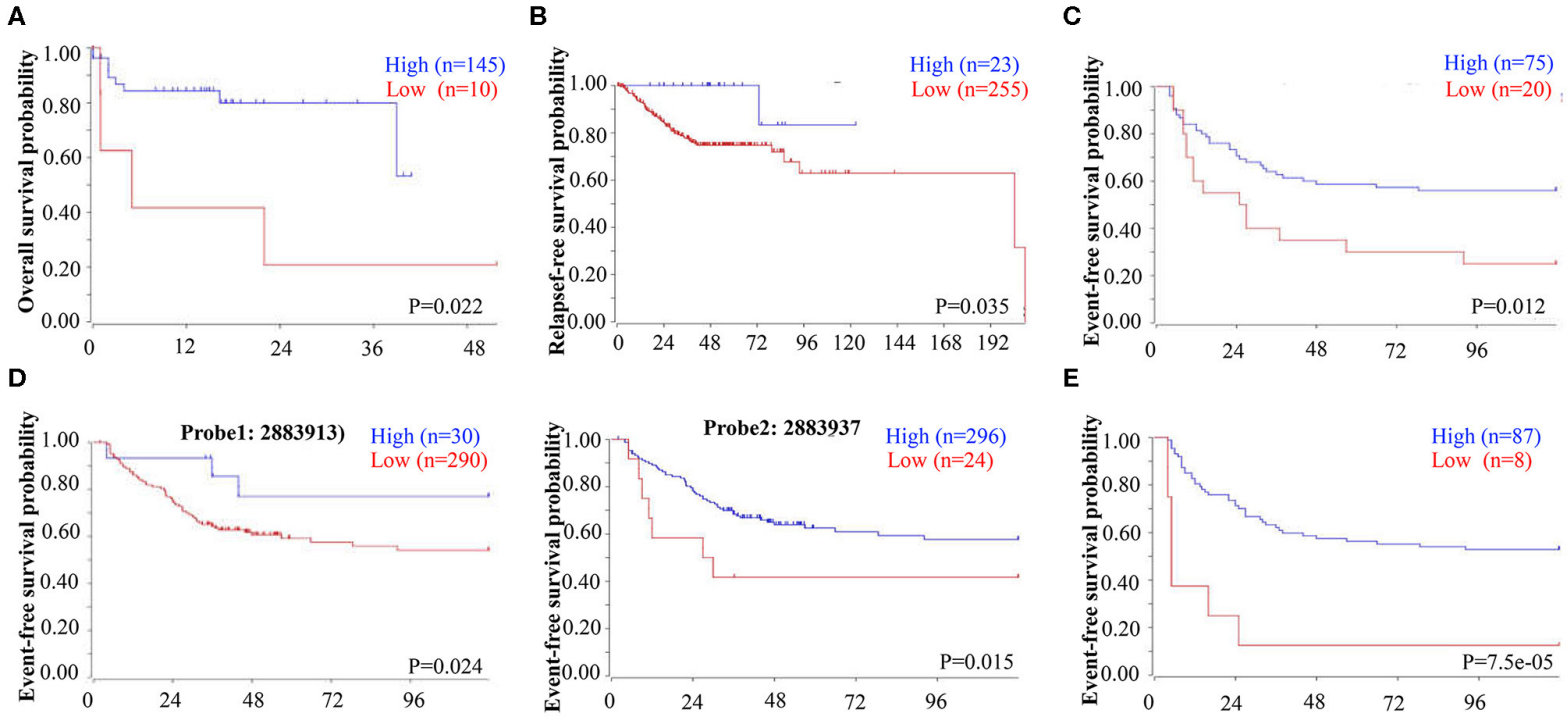
Low EBF1 expression correlates with shorter survival in CRC patients. **(A–E)** A public clinical microarray dataset from the R2 bioinformatic platform was used to analyze the correlation between EBF1 mRNA expression and overall survival **(A)**, relapse-free survival **(B)**, and event-free survival **(C–E)** of CRC patients.

To determine whether decreased EBF1 levels might have a biologic effect in CRC, we next determined the relationship between decreased EBF1 mRNA levels and survival of CRC patients. Analyses of different datasets within the R2 website demonstrated that CRC patients with lower mRNA levels of EBF1 have shorter overall survival (Figure 2A), relapse-free survival (Figure 2B), and event-free survival (Figures 2C–E) compared to those with high EBF1 levels. These results suggest that low expression of EBF1 in CRC plays a role in the pathophysiology of the disease. Thus, EBF1 might be involved in the development and progression of CRC and be a predictor of shorter survival.

### EBF1 Over-Expression Suppresses CRC Cell Growth *in vitro* and *in vivo*

**Figure 3:**
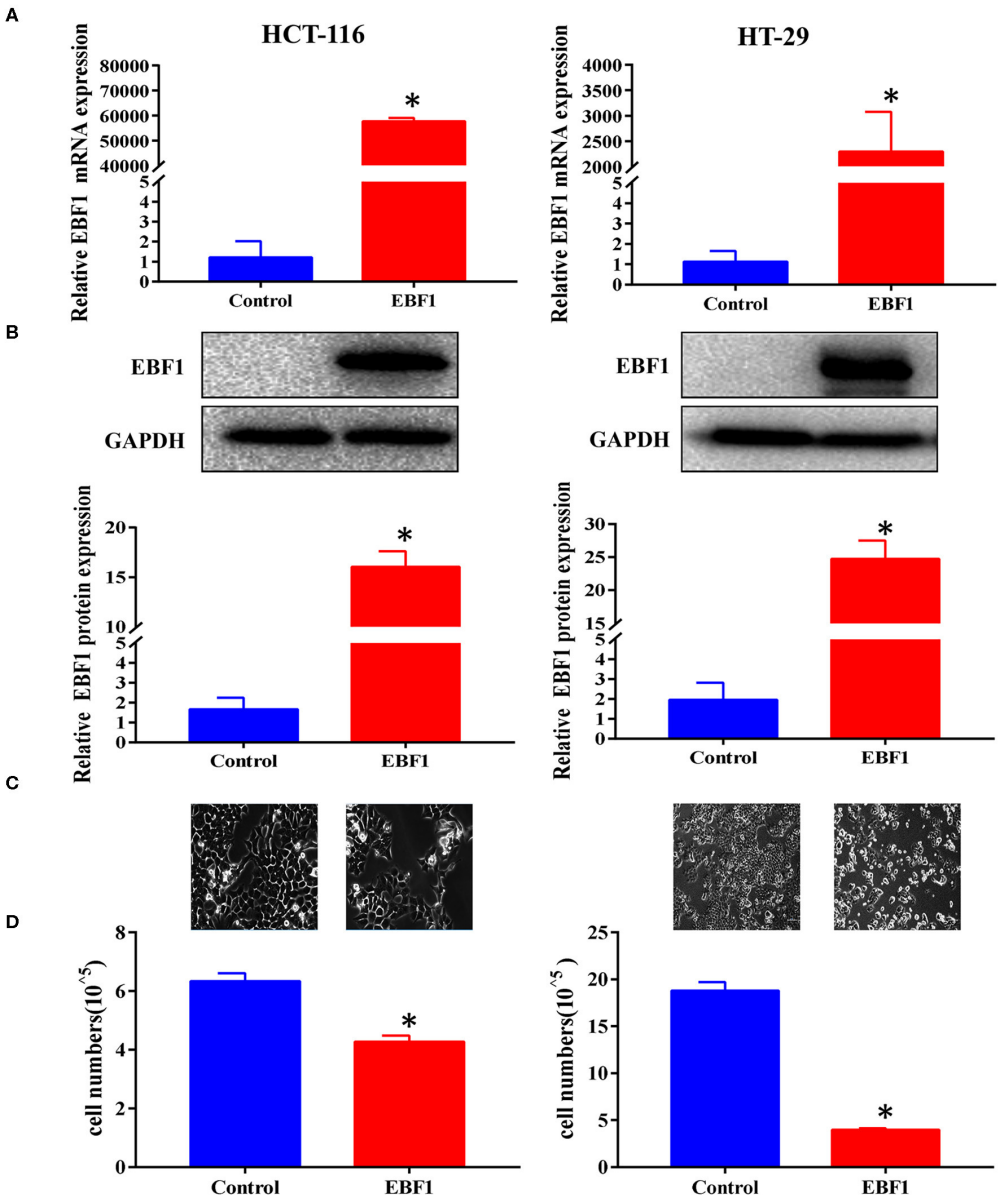
EBF1 over-expression suppresses CRC cell growth *in vitro*. **(A,B)** HCT-116 and HT-29 cells were stably transduced with EBF1 or control. The cells were evaluated at a fixed time in culture. **(A)** Q-PCR and **(B)** western blot were performed to determine the expression of EBF1. GAPDH was used as an internal control. **(C)** Cell confluence of transduced HCT-116 and HT-29 cells were imaged using a phase contrast fluorescence microscope in light at a magnification of 200×. **(D)** Cell number was determined with a Countstar Automated Cell Counter. *P < 0.05 vs. control.

**Figure 4:**
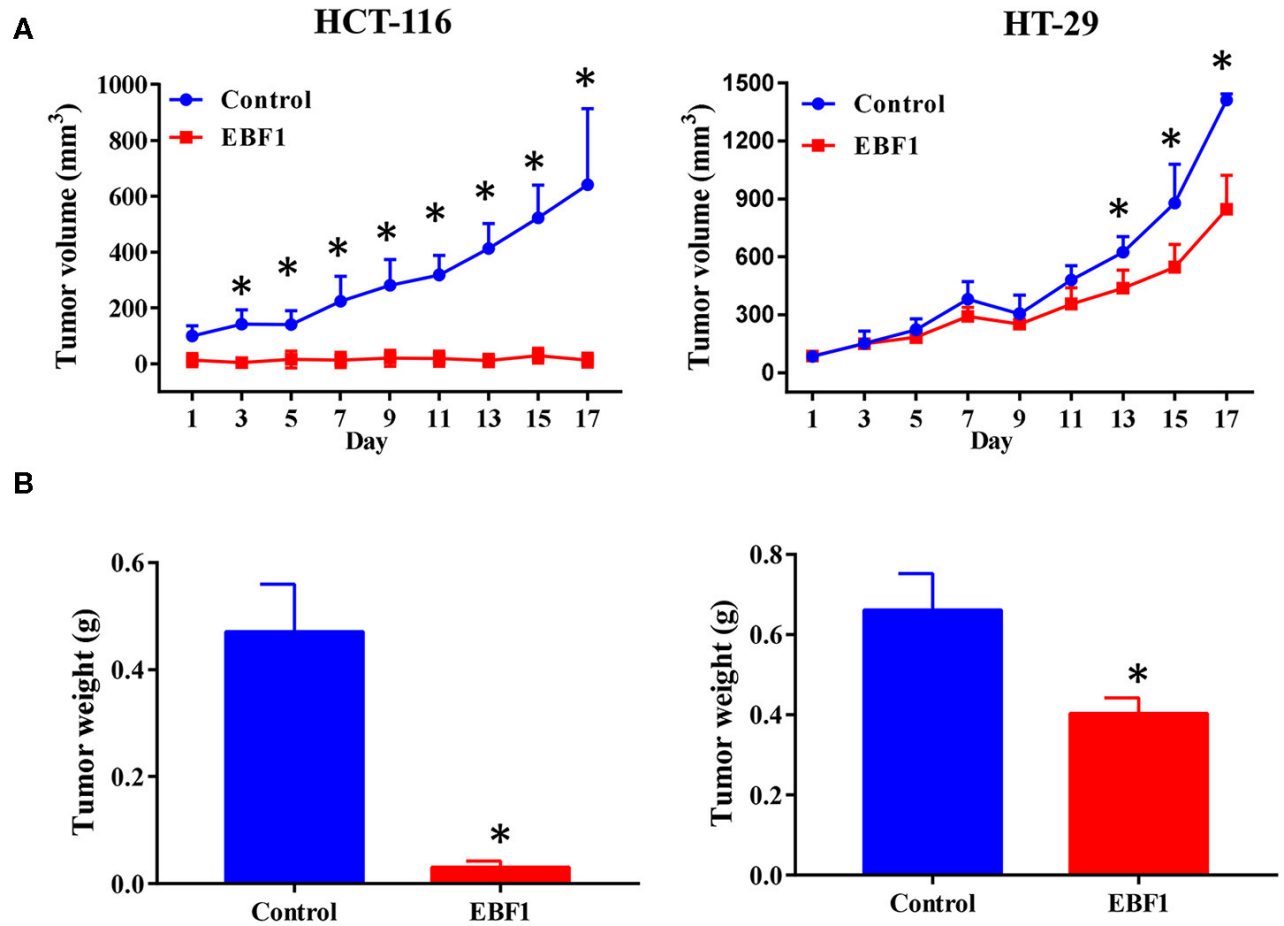
EBF1 over-expression suppresses CRC cell growth *in vivo*. Stably transduced HCT-116 and HT-29 cells over expressing EBF1 or control were injected subcutaneously into BALB/C nude mice. **(A)** Tumor volume was measured on the indicated days and **(B)** tumor weight was determined at the end of experiments. *P < 0.05 vs. control.

To further assess the biological function of EBF1 in CRC, cultured CRC (HCT-116 and HT-29) cells were transduced with a lentivirus encoding EBF1 over-expression or control plasmid. QPCR and western-blot analyses demonstrated that transduction of EBF1 over-expression plasmid increased the expression of EBF1 at both mRNA (Figure 3A) and protein levels (Figure 3B). Observation of cell morphology and counting of cell number showed that EBF1 over-expression significantly reduced cell confluence (Figure 3C) and cell number in CRC cells (Figure 3D). To investigate whether the effect of EBF1 on CRC cell growth occurred *in vivo*, EBF1-over-expressing HCT116 and HT-29 cells or control cells (1 × 10^6^ cells per injection site) were implanted into male nude mice. In both cell lines, tumor growth was attenuated in the mice implanted with the EBF1 over-expressing cells (Figure 4) These studies indicate that EBF1 over-expression significantly suppresses tumor growth *in vitro* and *in vivo*.

### EBF1 Over-Expression Inhibits Proliferation and Induces Apoptosis of CRC Cells

**Figure 5:**
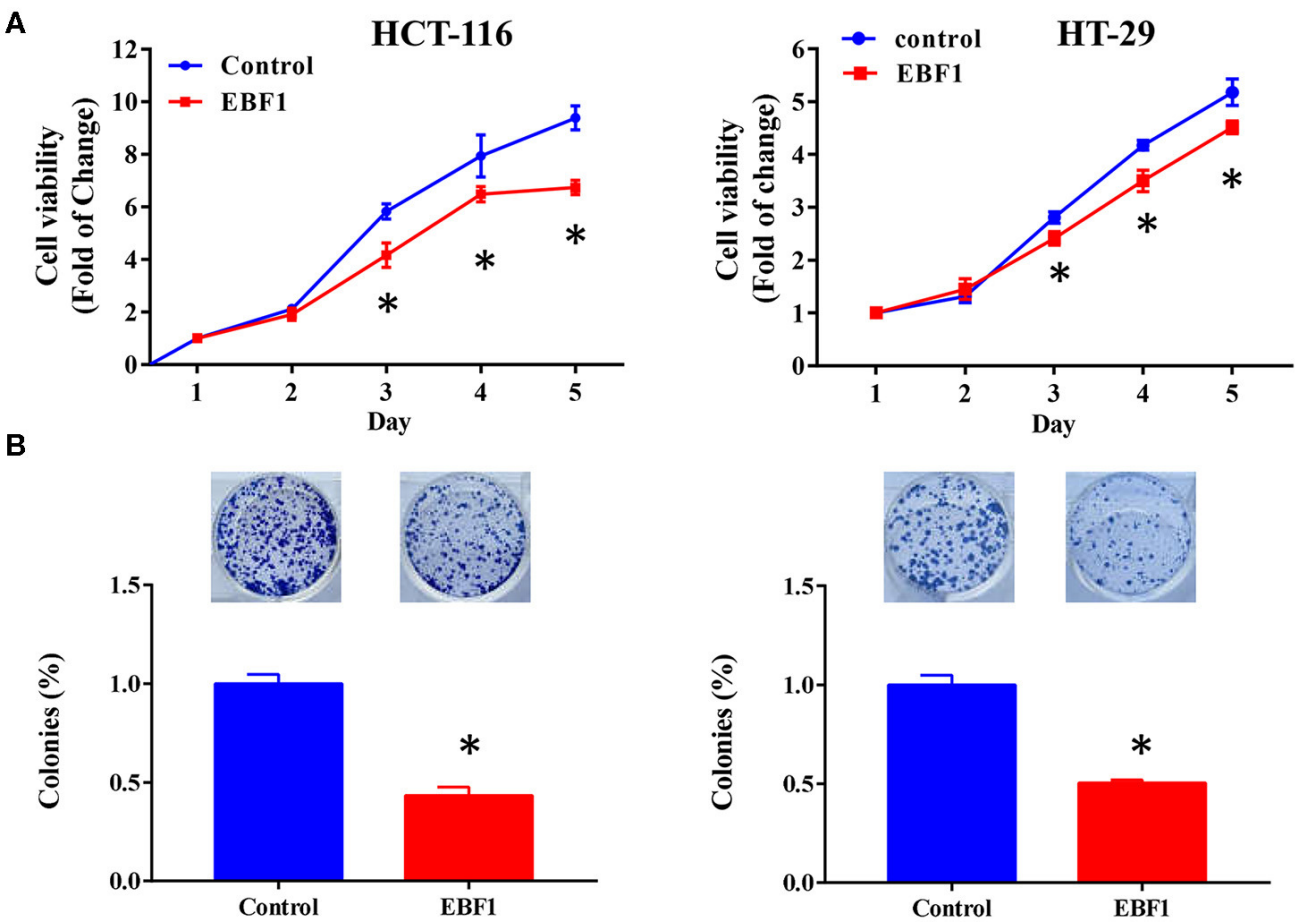
EBF1 over-expression reduces cell viability and cell survival in CRC cells. **(A)** Cell count assay was performed to determine the cell viability of HCT-116 and HT-29 cells. Cell viability was normalized to the OD value of Day 1 for each group. **(B)** Colony formation assay was used to determine survival of HCT-116 and HT-29 cells; survival rates were normalized to the number of colonies in the control (100%) group, *P < 0.05 vs. control.

**Figure 6:**
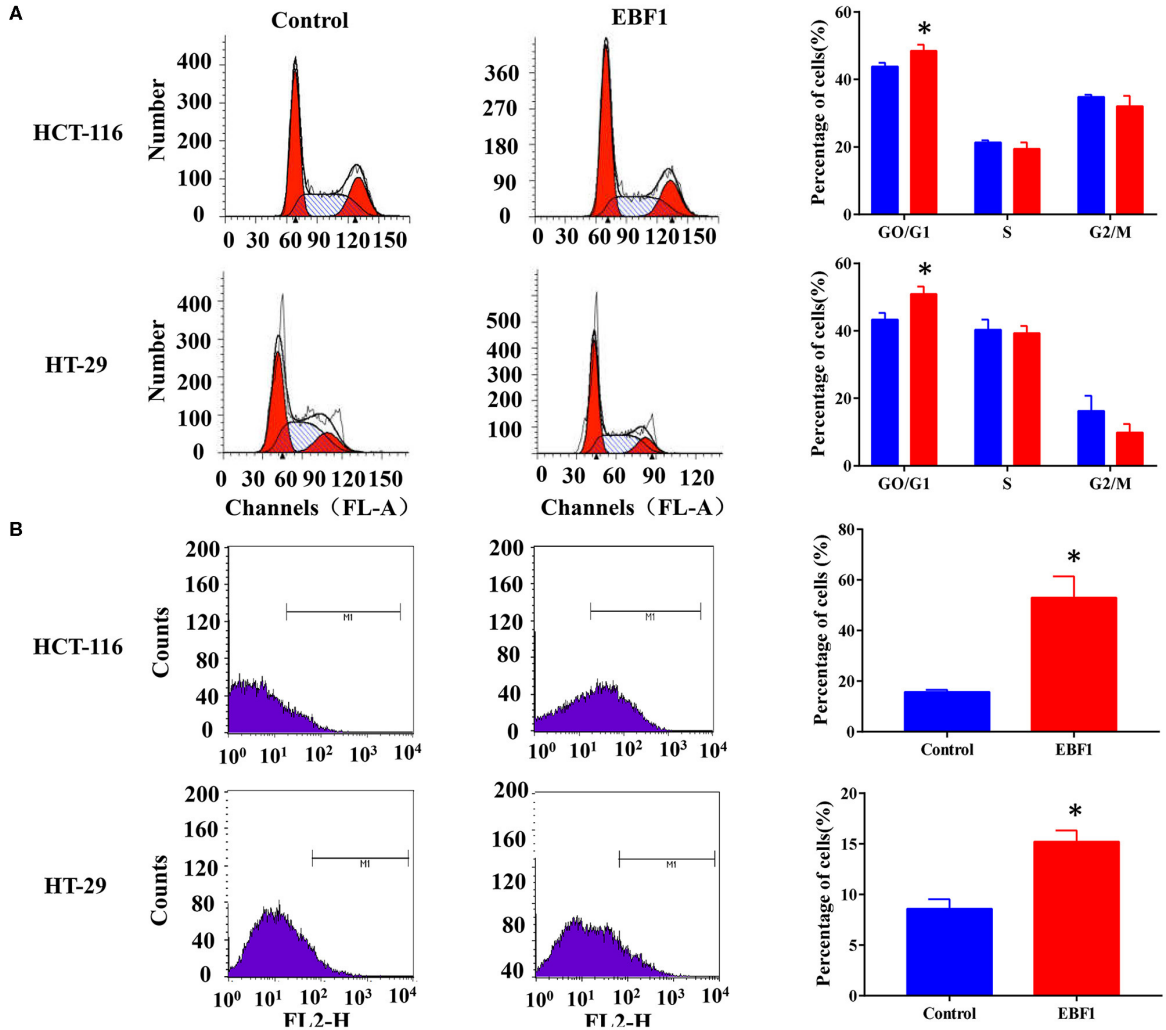
EBF1 over-expression induces cell cycle arrest and cell apoptosis in CRC cells. **(A)** Propidium iodide staining and flow cytometry analysis were performed to determine cell cycle progression of HCT-116 (upper panel) and HT-29 (lower panel) cells after EBF1 over-expression. The representative images (left panel) and analysis of cell cycle distributions (right panel) are shown. **(B)** Annexin V staining and flow cytometry analysis were performed to measure apoptosis of HCT-116 (upper panel) and HT-29 (lower panel) cells after EBF1 over-expression. Representative images (left panel) are presented and the percentages of apoptotic cells calculated (right panel). *P < 0.05 vs. control.

To further evaluate the functional role of EBF1 in CRC, we investigated the effect of EBF1 over-expression on cell proliferation and apoptosis. CCK-8 and colony formation analyses revealed that EBF1 over-expression significantly decreased cell viability (Figure 5A) and colony formation (Figure 5B) in both HCT116 and HT-29 cells. Cell cycle analysis showed that EBF1 over-expression increased the percentage of cells in the G0/G1 phases (Figure 6A). Moreover, cell apoptosis analysis using annexin V staining indicated that EBF1 over-expression increased the percentage of apoptotic cells (Figure 6B). These studies are consistent with our previous work (26) and suggest a tumor suppressor role for EBF1 in CRC.

### EBF1 Over-Expression Down-Regulates PNO1 and Up-Regulates p53 and p21 Expression

**Figure 7:**
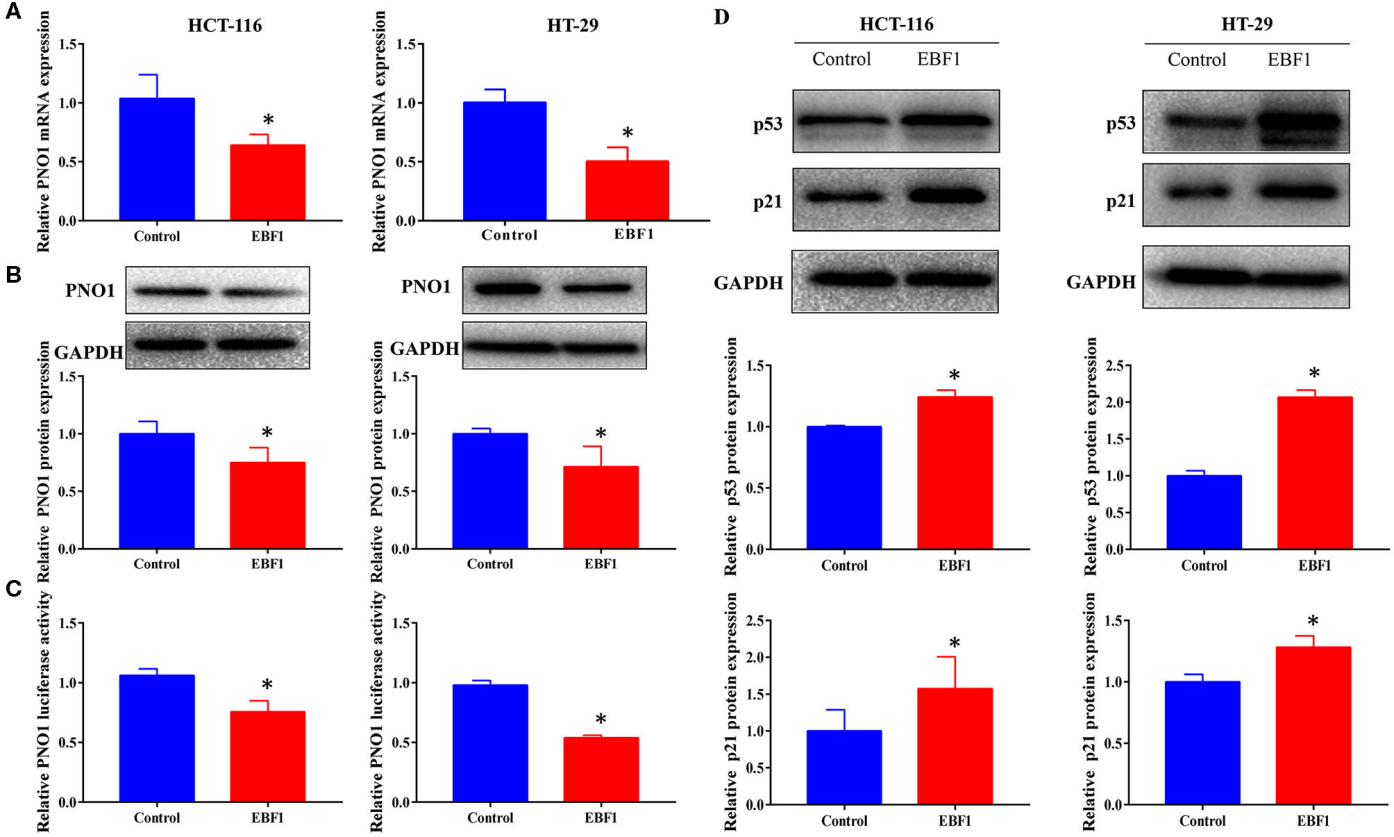
EBF1 over-expression down-regulates PNO1 and up-regulates p53 and p21 expression. **(A,B)** HCT-116 and HT-29 cells were stably transduced with EBF1 or control plasmid, and **(A)** Q-PCR and **(B)** western blotting were performed to determine the expression of PNO1, GAPDH was used as a loading control. **(C)** Luciferase assay was performed to determine the effect of EBF1 over-expression on PNO1 transcription in both HCT-116 and HT-29 cells. **(D)** Western blotting was performed to determine the protein expression of p53 and p21 in HCT-116 and HT-29 cells after EBF over-expression. GAPDH was used as a loading control. *P < 0.05.

Our previous study identified EBF1 as an upstream transcription factor of the potential oncogene PNO1. We confirmed that EBF1 over-expression down-regulated PNO1 expression on both mRNA and protein levels (Figures 7A,B). Moreover, EBF1 overexpression decreased PNO1 promoter activity in both HCT116 and HT-29 cells as determined using a PNO1-luciferase reporter system (Figure 7C). Since PNO1 promotes CRC cell growth by negatively regulating the activation of p53/p21 pathway (26), we further determined the expression of p53 and p21 by western-blot analysis. We found that EBF1 over-expression significantly increased p53 and p21 protein expression in both HCT116 and HT-29 cells (Figure 7D). Taken together, these results indicate that EBF1 might act as a tumor suppressor by negatively down-regulating PNO1 expression and activating the p53/p21 pathway.

## Discussion

In this study we report that the transcription factor EBF1 is down-regulated in CRC tissues of patients with poorer outcomes and that EBF1 over-expression suppresses tumor growth *in vivo* and *in vitro* by inhibiting proliferation and inducing apoptosis. Moreover, EBF1 over-expression significantly down-regulates PNO1 and increases p53 and p21 expression. These findings lead us to propose a tumor suppressor role for EBF1 in CRC.

Recent studies in BC, PDAC, and CCA demonstrate lower EBF1 expression in tumor tissues compared to related non-cancerous tissues (22, 24, 25). Similarly, in this study we show that EBF1 mRNA levels are decreased in CRC tissues suggesting that decreased EBF1 expression might play an essential role during development of CRC. Moreover, consistent with survival analysis in CCA patients (25), we found that low EBF1 expression was correlated with shorter overall, remission-free, and event-free survival in CRC patients. These results provide evidence that EBF1 could be a potential prognostic biomarker and/or molecular target for CRC. However, the protein expression of EBF1 and its correlation with survival in CRC patients should be further explored.

Loss of EBF1 contributes to the development of B-ALL (16–19) and over-expression of EBF1 increases DNA damage in a dose-dependent manner (20), indicating a tumor suppressor role for EBF1 in leukemia. An investigation of CCA demonstrated that siRNA-mediated EBF1 knockdown increased cell viability, wound healing, and cell migration of CCA cells (25). Moreover, our previous study indicated that EBF1 over-expression suppresses cell growth *in vitro* (26). Our current results further confirm the these findings. Specifically, we demonstrate that EBF1 over-expression markedly inhibited CRC cells growth and viability *in vitro* and *in vivo*. Furthermore, EBF1 over-expression induced cell cycle arrest in the G0/G1 phase and increased apoptosis. These results further support a tumor suppressor role for EBF1 in CRC. One potential shortcoming in the current study is the use of lentivirus-based overexpression leading to levels of EBF1 which are potentially higher than endogenous levels in normal colon tissue. Some studies indicate that high levels of protein may cause overexpression toxicity that can non-specifically harm cells in several ways. For example, they may over activate specific biological pathways and disrupt regulation of downstream partners (33–35). Future studies should compare the expression of EBF1 in normal intestinal epithelial cells (FHC) with CRC cell lines. Furthermore, inducible EBF1 overexpression techniques can be used to confirm an expression-dependent tumor suppressor role for EBF1 in CRC.

As a transcript factor, EBF1 over-expression increases DNA damage by directly targeting RAD51 in leukemia (20), while EBF1 knockdown promotes cell proliferation and migration by increasing CD133, Oct3/4, and TFF1 expression in CCA (25). In the present study, we confirmed that EBF1 over-expression decreased mRNA and protein levels, as well as the promoter activity of PNO1 in both HCT116 and HT-29 cells. These findings are consistent with our previous study in RKO cells (26). To test whether EBF1 binds the PNO1 gene promoter, analysis of datasets within the ChIP-seq and JASPAR websites was performed. The results suggest that EBF1 binds the promoter region of the PNO1 gene (from approximately −850 to −950). However, the interaction of EBF1 with the promoter region of PNO1 will need to be further validated by CHIP and EMSA assays. As a down-stream target of EBF1, PNO1 promotes cell proliferation by negatively regulating p53 and p21 (26). We therefore determined the activation of p53/p21 pathway in CRC cells after EBF1 over-expression. As hypothesized, EBF1 over-expression significantly increased the protein expression of p53 and p21 in CRC cells. These findings preliminarily suggest that down-regulation of PNO1 expression mediates the activation of the p53/p21 signaling pathway, and might be one of the mechanisms underlying EBF1-mediated tumor growth suppression. However, more in-depth mechanistic research including rescue assays to confirm the role of EBF1 in PNO1/P53/P21 pathway are needed and the effect of EBF1 over-expression on activation of other signaling pathways should be further addressed.

In summary, we report that low EBF1 expression levels in CRC correlates with shorter survival of patients and that EBF1 over-expression suppresses tumor growth by inhibiting cell proliferation and inducing cell apoptosis in CRC cells. These effects result, in part, from the inhibition of PNO1-mediated p53/p21 signaling pathway activation.

## Data Availability Statement

The raw data supporting the conclusions of this article will be made available by the authors, without undue reservation, to any qualified researcher.

## Ethics Statement

This animal study was reviewed and approved by Institutional Animal Care and Use Committee at the Fujian University of Traditional Chinese Medicine.

## Author Contributions

AS and JP conceived and designed the experiments. ZS, LLiu, XW, and XL conducted bioinformatics analysis and cDNA array analysis. ZS, MP, LLi, and XC conducted analyses of cell viability and cell survival. YChen and LLi conducted cell cycle and cell apoptosis analyses. AS, XW, MW, and LLi performed the animal experiments. MP, YCheng, and XC conducted western blot. LLiu, AS, JC, and ZS performed the data analysis. AS, LLi, and ZS produced the figures. TS and JP reviewed the data and revised the manuscript. ZS, YChen, JC, and AS wrote the manuscript. All authors contributed to the article and approved the submitted version.

## Conflict of Interest

The authors declare that the research was conducted in the absence of any commercial or financial relationships that could be construed as a potential conflict of interest.
